# Anterior knee pain is a relevant cause for dissatisfaction after total knee arthroplasty without patellar resurfacing

**DOI:** 10.1002/jeo2.70382

**Published:** 2025-08-05

**Authors:** Jörg Lützner, Patrick Schubert, Franziska Beyer, Cornelia Lützner

**Affiliations:** ^1^ University Center for Orthopaedics, Trauma and Plastic Surgery (OUPC), University Hospital Carl Gustav Carus Technische Universität Dresden Dresden Germany; ^2^ Center for Evidence‐Based Healthcare (ZEGV), University Hospital Carl Gustav Carus and Medical Faculty Technische Universität Dresden Dresden Germany

**Keywords:** anterior knee pain, knee arthroplasty, outcome, pain, patellar resurfacing, PROM

## Abstract

**Purpose:**

The aim of the present study was to investigate the incidence of anterior knee pain (AKP) after total knee arthroplasty (TKA) without patellar resurfacing and to determine how patient‐reported outcome measures (PROMs) differ in patients with and without AKP.

**Methods:**

All primary TKAs from the local registry operated on between 2010 and 2016 were reviewed for secondary patellar resurfacing and asked about the presence of AKP (yes/no), satisfaction with the outcome of the TKA (Visual Analogue Scale [VAS] 1–10) and completion of the Oxford Knee Score (OKS) and Anterior Knee Pain Scale (AKPS). A total of 1371 primary TKAs were included, of which 201 patients had died, and 972 (70.9%) completed the additional questionnaires after a mean follow‐up of 9.9 years.

**Results:**

AKP was reported by 157 patients (16.2%), and eight patients (0.6%) had undergone secondary patellar resurfacing within two to five years after primary TKA, two of them with an additional increase of the insert due to instability. Patients who reported AKP had significantly worse OKS (mean 24.0 vs. 39.6), AKPS (mean 50.8 vs. 79.1) and satisfaction (mean 4.8 vs. 8.4/10). There were no statistically significant differences in the incidence of AKP by sex (men 15.1% vs. women 16.4%), age at surgery (<60 years 19.6% vs. ≥60 years 15.2%), comorbidities (ASA 1/2 14.5% vs. ASA 3/4 18.1%) and constraint (unconstrained 15.9% vs. rotating‐hinge 26.3%). The incidence of AKP in unconstrained TKA was 16.0% in CR, 12.6% in UC and 18.5% in PS implants. AKP occured significantly more frequently in patients with a BMI > 40 kg/m^2^ (26.0%, *p* = 0.03). In a multivariate regression analysis, only preoperative OKS had an independent influence on AKP (OR 0.949 per point, *p* = 0.003).

**Conclusion:**

In this study with long‐term follow‐up, a relevant proportion of patients reported AKP, which had a negative impact on PROMs and satisfaction. However, the rate of secondary patellar resurfacing was very low.

**Level of Evidence:**

Level III, therapeutic study.

**Abbreviations**
AKP: anterior knee pain
AKPS: Anterior Knee Pain Scale
AKSFS: American Knee Society Function Score
AKSKS: American Knee Society Knee Score
ASA: American Society of Anesthesiologists
BMI: body mass index
CCK: condylar constraint
CI: confidence interval
CR: cruciate retaining
FU: follow‐up
IQR: interquartile range
KOOS: Knee Injury and Osteoarthritis Outcome Score
KSS: Knee Society Score
LEFS: Lower Extremity Functional Scale
NICE: National Institute of Health and Care Excellence (NICE)
NJR: National Joint Registry
OKS: Oxford Knee Score
OR: odds ratio
PCL: posterior cruciate ligament
PD: patellar denervation
PFS: Patellar Feller score
PND: patellar non‐denervation
PNR: patella non‐resurfacing
PR: patella resurfacing
PROMs: patient‐reported outcome measures
PS: posterior stabilised
RCT: randomised controlled trial
ROM: range of motion
TKA: total knee arthroplasty
UC: anterior‐lipped ultracongruent
VAS: Visual Analogue Scale

## INTRODUCTION

Anterior knee pain (AKP) is a frequent problem after total knee arthroplasty (TKA), that occurs in the anterior and central aspects of the knee and is associated with several functional problems [9]. In some cases, AKP may be caused by distinct mechanical or functional problems, but usually the underlying causes remain ambiguous. This presents a challenge for patients and surgeons, as a revision is often unsuccessful without a clear reason [33].

Various intraoperative strategies are used to avoid AKP, including patellar denervation, patellar‐friendly femoral components or patellar resurfacing. Patellar resurfacing has been widely studied but is still controversial [7, 23, 24]. The rates of patellar resurfacing vary considerably across arthroplasty registries worldwide [19]. According to the latest reports, the rates and trends are as follows: USA 88.6%, slightly decreasing [1], Australia 78.1%, increasing [25], Netherlands 14.9%, decreasing [8], Germany 10.5%, slightly decreasing [12] and Sweden 4.0%, slightly increasing [27]. The National Institute of Health and Care Excellence (NICE) has recommended that patellar resurfacing should be offered for all primary TKAs. However, this recommendation was based on ‘… not enough clinical evidence …’, but ’… strong economic evidence …’ [17]. At a recent international consensus conference (World Expert Meeting in Arthroplasty 2024), it was recommended that selective patellar resurfacing appears justified, which was based on the results of an updated meta‐analysis [16].

AKP has been assessed heterogeneously, either using a Visual‐Analogue Scale (VAS), single‐item questions regarding the presence or absence of AKP, or a selection of items indicating patellofemoral problems derived from validated patient‐reported outcome measures (PROMs). The Anterior Knee Pain Scale (AKPS, Kujala‐Score) and the Lower extremity functional scale (LEFS) are the two recommended validated PROMs to exclusively measure pain and function in individuals with AKP [32].

Although there are many studies regarding patellar problems and resurfacing, there is no clear data on how frequently AKP occurs after TKA. Therefore, the aims of the present study were to investigate the incidence of AKP after TKA without patellar resurfacing, to determine how PROMs differ in patients with and without AKP, and to investigate the association between different patient and implant factors with AKP. We hypothesised that PROMs and satisfaction would be worse in patients with AKP.

## MATERIALS AND METHODS

### Study design and setting

The Dresden Hip and Knee Registry was established in 2005 and ethical approval was granted from the institutional review board (EK 135042014). It is a single center registry at a university hospital. All patients scheduled for a hip or knee arthroplasty are invited to participate and must sign an informed consent form. Patients complete a standard follow‐up protocol with a clinical examination 3 months and a postal survey 12 months after surgery.

### Study population

**Figure 1:**
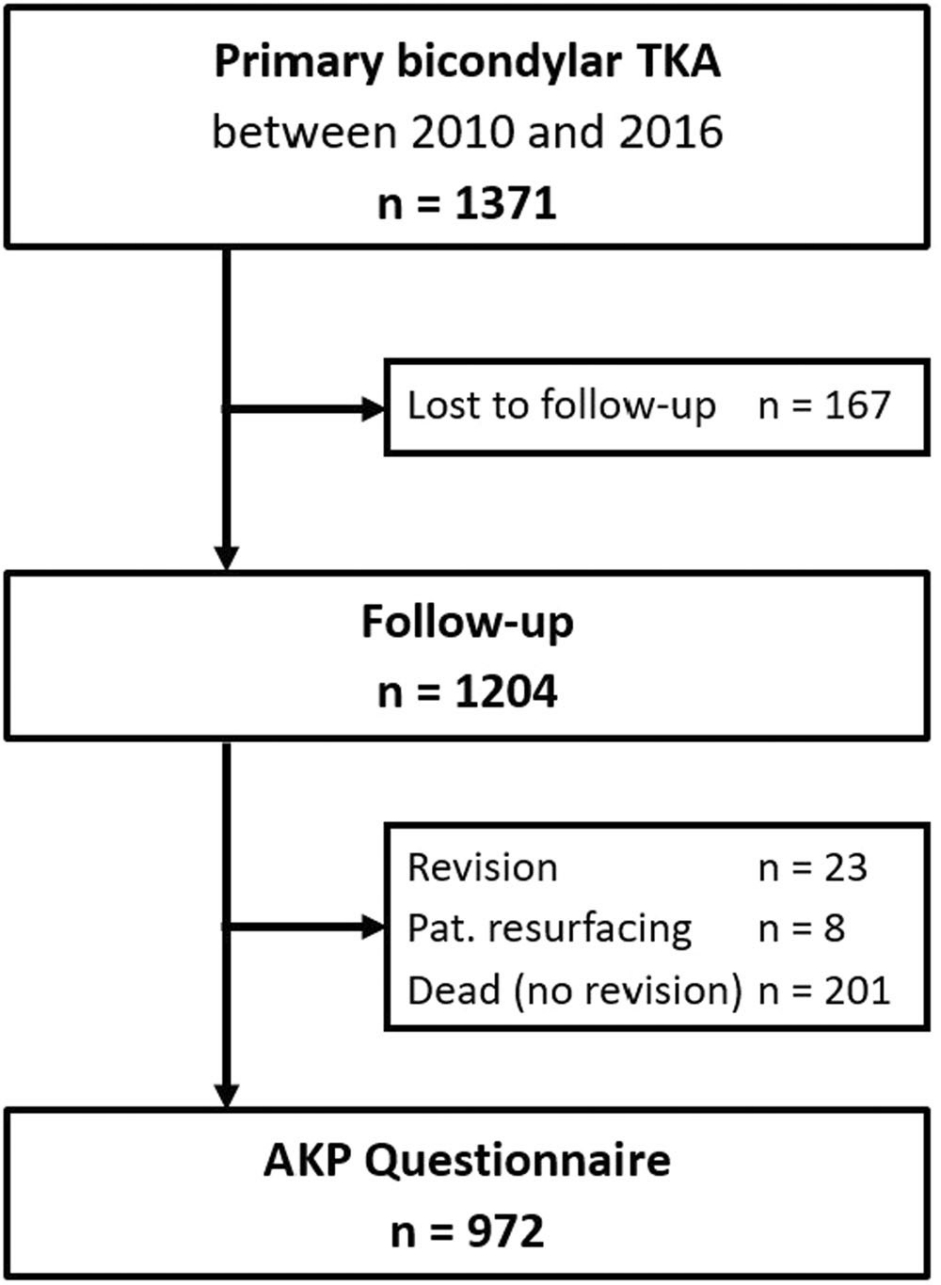
Flowchart of study patients. AKP, anterior knee pain; TKA, total knee arthroplasty.

For this prospective study, all primary TKAs operated on between 2010 and 2016 were identified and asked to participate in a further follow‐up. Partial knee replacements (unicondylar and patellofemoral) were excluded. Of a total of 1371 primary TKAs, 1204 were available for further follow‐up (Figure 1). There were 23 revisions and additional eight secondary patellar resurfacing, 201 patients had died without revision, and 972 (70.9%) completed the questionnaire after an average of 9.9 years (range 6.5–13.5). These 972 patients had a mean age of 68.3 years (SD 9.4), a mean BMI of 31.0 kg/m^2^ (SD 5.6), and 61.2% were women.

A variety of implants have been used that can be considered ‘patella‐friendly’ according to published criteria [18, 24]. Apart from the implants, whose use has changed over time and according to surgeon preference, the surgical procedure was well standardised. All surgeries were performed via medial parapatellar approach aiming for a mechanical alignment. All components were cemented. Patellar denervation and lateral facetectomy were performed, but no patellar resurfacing. Non‐resurfacing was systematically performed regardless of the cartilage damage of the patella. Cruciate‐retaining (CR) inserts were preferably used. If the posterior cruciate ligament (PCL) was not intact or needed to be resected, anterior‐lipped ultracongruent (UC) or posterior‐stabilised (PS) inserts were used, according to the surgeon's preference.

### Outcome measures

As part of the registry, patients completed PROMs before as well as 3 and 12 months after surgery. For this study, all available patients received a postal survey and completed questionnaires consisting of the Oxford Knee Score (OKS), the AKPS, a single‐item question on the presence of AKP (yes/no), and a question on overall satisfaction with the outcome of the TKA (VAS 0–10).

To ensure correct allocation, patients who reported at least moderate pain in the OKS (item no. 1, usual knee pain) or AKPS (item no. 9, knee pain in general) were asked again by telephone about the specific location of the pain. If the patients indicated the pain location as anterior or answered the question about AKP in the affirmative, they were allocated to the AKP group.

In addition, the rates of secondary patellar resurfacing, the estimates of all‐cause revision‐free survivorship and patellar revision free survivorship were calculated.

### Data analysis

Data description was based on medians and interquartile range (IQR) for continuous values and absolute and relative frequencies for categorical values. Comparisons between groups were carried out with Mann–Whitney *U* test for continuous values when the Kolmogorov–Smirnov test indicated non‐normally distributed data, and the chi‐square test for categorical values. Implant survival was calculated using Kaplan–Meier survival analysis. Univariate and multivariate regression analysis were performed to determine the influence of different patient and implant factors on AKP. SPSS software (release 29 for Windows by IBM Corp., Armonk, New York, USA) was used for data analysis.

## RESULTS

**Figure 2:**
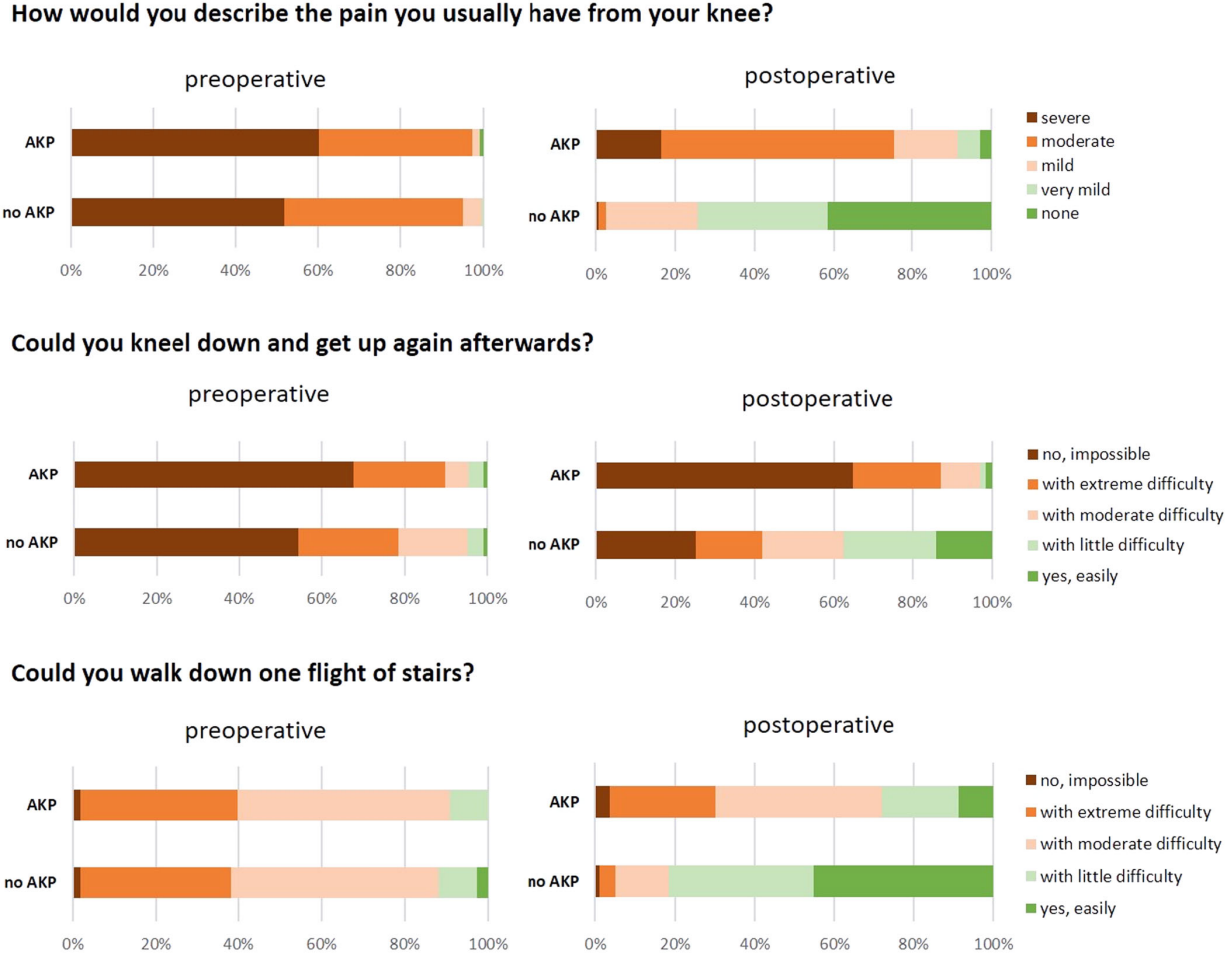
Items of patella‐related activities of the OKS in patients with and without AKP. AKP, anterior knee pain; OKS, Oxford Knee Score.

AKP was reported by 157 of the 972 patients (16.2%) according to the applied definition. Baseline data distributed for patients with and without AKP are displayed in Table 1, and there were no significant differences between the groups. Patients who reported AKP showed significantly worse PROMs (OKS and AKPS) and satisfaction with the outcome of the TKA (Table 1). While the OKS improved substantially in patients without AKP, there was only a small increase in patients with AKP. In the OKS, patients with AKP indicated significantly more pain (*p* < 0.001) and difficulty with patella‐related activities (kneeling down and getting up, walking down stairs, *p* < 0.001) than patients without AKP (Figure 2).

**Table 1:** Baseline data and PROMs for patients with and without AKP given as median (IQR) or as absolute and relative frequencies.

Anterior knee pain (AKP)	No 83.8%	Yes 16.2%	*p* value
Age at surgery (years)	69.8 (62.3–75.0)	68.3 (59.6–74.8)	0.123
Sex			
Women	496 (60.9%)	99 (63.1%)	0.605
Men	319 (39.1%)	58 (36.9%)	
BMI (kg/m^2^)	30.0 (27.0–34.1)	30.9 (27.8–35.2)	0.076
ASA grade			
1/2	449 (55.1%)	76 (48.4%)	0.086
3/4	366 (44.9%)	81 (51.6%)	
Follow‐up (years)	10.3 (8.4–12.1)	9.5 (8.2–11.9)	0.074
AKPS (0–100)			
Latest follow‐up	80.0 (70.0–90.0)	49.0 (42.0–60.0)	**<0.001**
Oxford Knee Score (0–48)			
Preoperative	23.0 (19.0–27.0)	20.0 (15.0–25.0)	**<0.001**
Latest follow‐up	41.0 (36.0–45.0)	24.0 (19.0–29.0)	<**0.001**
Satisfaction with the outcome of the TKA (0–10)	9.0 (7.5–9.5)	5.0 (3.0–6.5)	<**0.001**

Abbreviations: AKPS, Anterior Knee Pain Scale (Kujala Score); ASA, American Society of Anesthesiologists; BMI, body mass index; IQR, interquartile range; PROMs, patient‐reported outcome measures; TKA, total knee arthroplasty.

The univariate analysis of different sociodemographic and implant factors is displayed in Table 2. There were no statistically significant differences in the incidence of AKP by sex (men 15.1% vs. women 16.4%), age at surgery (<60 years 19.6% vs. ≥60 years 15.2%), comorbidities (ASA 1/2 14.5% vs. ASA 3/4 18.1%) and constraint (unconstrained 15.9% vs. rotating‐hinge 26.3%). The incidence of AKP in unconstrained TKA was 16.0% in CR, 12.6% in UC and 18.5% in PS implants. In a multivariate regression analysis (Table 3), only the preoperative OKS (OR 0.949, 95%CI 0.916–0.982, *p* = 0.003) had an independent significant influence on AKP. Each additional point in the preoperative OKS resulted in 5% lower risk of reporting AKP at the latest follow‐up.

**Table 2:** Rates of anterior knee pain, given as absolute and relative frequencies.

Anterior knee pain (AKP)	No *N* (%)	Yes *N* (%)	*p* value
Age at surgery			
<60 years	164 (80.4)	40 (19.6)	0.105
60–70 years	249 (83.6)	49 (16.4)	
>70 years	402 (85.5)	68 (14.5)	
ASA grade			
1	42 (95.5)	2 (4.5)	0.063
2	407 (84.6)	74 (15.4)	
3	363 (81.8)	81 (18.2)	
4	3 (100)	n.a.	
BMI			
<30	410 (86.1)	66 (13.9)	**0.030**
30–35	237 (82.6)	50 (17.4)	
35–40	114 (83.8)	22 (16.2)	
>40	54 (74.0)	19 (26.0)	
Insert type			
CR	604 (84.0)	115 (16.0)	0.339
UC	97 (87.4)	23 (12.6)	
PS	101 (81.5)	14 (18.5)	
CCK	1 (100)	n.a.	
Rotating hinge	12 (70.6)	5 (29.4)	

Abbreviations: ASA, American Society of Anesthesiologists; BMI, body mass index; CCK, condylar constraint; CR, cruciate retaining; PS, posterior stabilised; UC, ultracongruent.

**Table 3:** Multivariate analysis of factors influencing AKP.

	OR	95% confidence interval OR		*p* value
		Lower	Upper	
Age at surgery [years]	0.985	0.960	1.011	0.250
BMI [kg/m²]	1.027	0.987	1.070	0.187
ASA 1/2 vs. 3/4	1.461	0.896	2.383	0.129
Sex [female]	0.876	0.548	1.400	0.581
Follow‐up [years]	1.032	0.937	1.137	0.519
Oxford Knee Score preoperative [points]	0.949	0.916	0.982	**0.003**
Constant	0.553			0.651

Abbreviations: AKP, anterior knee pain; ASA, American Society of Anesthesiologists; BMI, body mass index; OR, odds ratio.

**Figure 3:**
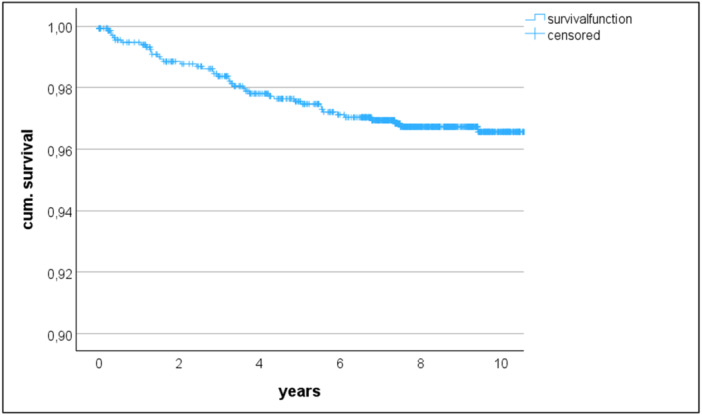
Kaplan–Meier survival analysis for all‐cause revision including secondary patellar resurfacing.

**Figure 4:**
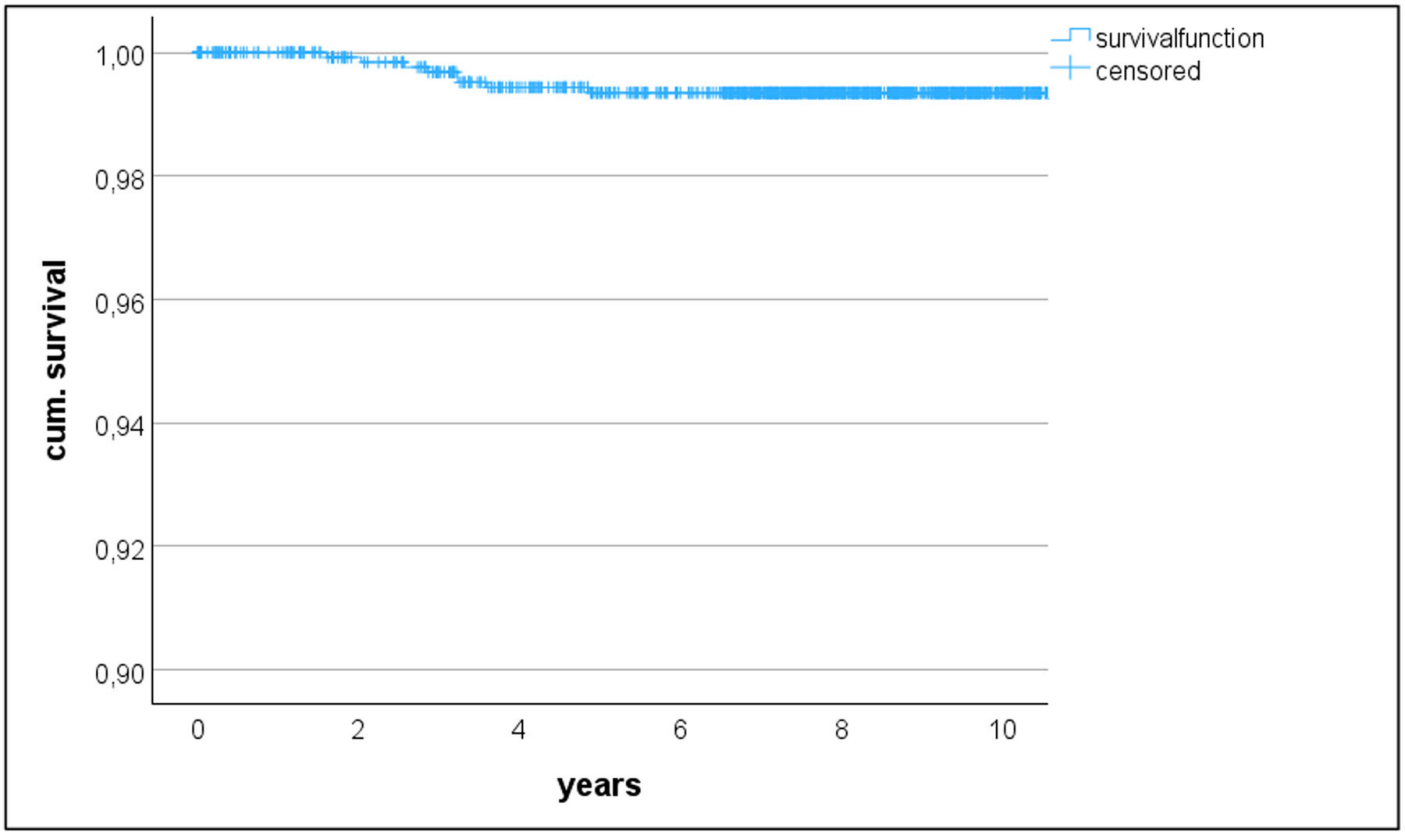
Kaplan–Meier survival analysis for secondary patellar resurfacing only.

A total of eight patients underwent secondary patellar resurfacing between two and five years after the initial surgery. Six of them for isolated AKP and two for AKP and instability with additional insert increase. The rate of secondary patellar resurfacing was therefore 0.6%, but accounted for 19% of all revisions. The 10‐year all‐cause revision‐free survivorship estimate was 96.6% (95%CI 95.3–97.5%) (Figure 3) and the estimate specifically related to the patella was 99.3% (95%CI 98.7–99.7%) (Figure 4).

## DISCUSSION

The main finding of this study was that AKP was associated with significantly worse PROMs. Persistent pain after TKA is still a frequent problem. In a systematic review of prospective studies on unselected osteoarthritis patients, Beswick et al. reported 10%–34% of patients with unfavourable pain after TKA [3]. A similar incidence of 16%–33% of chronic pain after TKA was reported in another review [33]. Pain is a subjective phenomenon and its localisation cannot always be reliably described. Despite these uncertainties, AKP is frequent and has been reported as one of the most common problems after TKA [20].

There are several recent meta‐analyses on patellar interventions during TKA surgery (patellar resurfacing, patellar denervation, infra‐patellar fat pad resection). The pooled data on AKP incidences ranged from 8% [13] to 22% [23] in TKA patients without patellar resurfacing (Table 4). These data must be evaluated cautiously as they were pooled across various follow‐up intervals, different implant designs and methods of measuring AKP. It needs to be acknowledged that most difficulties after TKA occur during patella‐related activities, and about 10%–20% of all patients will report some degree of AKP when specifically asked about it. In our cohort, 16.2% of patients reported AKP after TKA, resulting in significantly worse PROMs and lower satisfaction. Unfortunately, there is no simple solution to prevent this, as AKP is multifactorial [9, 29].

**Table 4:** Incidence rates of anterior knee pain in recent meta‐analysis.

Author	Studies	Incidence AKP	Conclusion
Chen et al. [4]	16 RCTs, 2013–2020	Patella resurfacing 11.9% Non‐resurfacing 19.9%	PR could reduce the occurrence of reoperation and noise after surgery, as well as increase the KSS total and function score, while it might not influence the outcomes such as AKP, ROM, OKS, KOOS, VAS, PFS, patellar tilt and patients'satisfaction
Feng et al. [10]	25 RCTs, 2002–2022	Patella resurfacing 16.6% Non‐resurfacing 20.3% Circumpatellar denervation 40.6% Non‐denervation 49.2%	PR, mobile‐bearing TKA, and fixed‐bearing TKA did not reduce incidence of AKP, lateral retinacular release can reduce AKP, whether PD can reduce AKP is controversial
Grela et al. [13]	33 RCTs, 5 observational studies, 1995–2022	Patella resurfacing 13.5% Non‐resurfacing 22.3%	PR reduced risk of AKP, revision surgery, and complications, no significant differences in PROMs (KSS, KOOS, OKS, PFS, VAS, ROM), supports primary TKA with PR over PNR
Kermanshahi et al. [16]	18 RCTs, 1996–2020	Patella resurfacing 10.2% Non‐resurfacing 19.7%	literature supports that PR during TKA results in fewer reoperations, PNR demonstrate non‐inferior clinical outcomes with better KSS total and functional scores at various FU, NPR was also associated with shorter operative times, selective PR appears justified, particularly in patients at risk of revision procedures
Qin et al. [21]	12 RCTs, 1 quasi‐RCT, 2004–2021	Patellar denervation 27.4% Non‐patellar denervation 37.1%	PD significantly reduces the AKP prevalence but not the AKP intensity following TKA, PROMs and function (ROM, AKSKS, AKSFS, PFS, OKS) may not be improved in PD compared to NPD after TKA, PD is preferred in both PR and PNR
Simpson et al. [24]	33 RCTs, 1983–2021	Patella resurfacing 11.2% Non‐resurfacing 18.2% Patella friendly: PR 12.6%, PNR 19.1% Non‐patellar friendly: PR 10.7%, PNR 17.9%	PR with a modern patellar friendly implant was not associated with lower rate of AKP, complications, reoperations, or significant differences in knee specific function compared to NPR; PR in combination with a non‐friendly TKA implant was associated with a significantly better OKS and lower reoperation rate
Simpson et al. [23]	33 RCTs, 1983–2022	Patella resurfacing 10.3% Non‐resurfacing 17.6% Subgroup CR insert: PR 8.2%, PNR 15.7% Subgroup PS implant: PR 12.7%, PNR 19.7%	PR, when performed with CR implants, resulted in lower rates of AKP and, when used with a PS implant, yielded better knee‐specific functional outcomes. Patellar resurfacing was associated with a lower risk of reoperation overall, but implant type did not influence this
Sun et al. [26]	9 RCTs, 2003–2017	Infra‐patellar fat pad resection 13.6% (until 1 year FU) Preservation 12.5% (until 1 year FU)	No statistical difference in AKP incidence, Insall‐Salvati ratio, and knee range of motion until 1 year FU
Tang et al. [28]	50 RCTs, 1989–2020	Patella resurfacing 16.2% Non‐resurfacing 18.1%	PR reduces revisions, AKP, and patellar clunk, results of PROMs lack clinical importance, PR resulted in higher KSS clinical scores, KSS functional scores, and OKS
Walker et al. [30]	5 RCTs, 5 pro‐, 3 retro‐spective studies, 2005–2023	Infra‐patellar fat pad resection 27.0% Preservation 21.1%	Rates of AKP at 6–12 months favoured preservation
Wang et al. [31]	9 RCTs, 2004–2015	Patellar denervation 38.8% (short‐term), 31.1% (long‐term) Non‐patellar denervation 49.3% (short‐term), 43.1% (long‐term)	PD significantly reduce incidence of AKP and VAS in FU within 12 months and improve the short‐term and long‐term knee function
Yuan et al. [34]	10 RCTs, 2004–2019	Patellar denervation 23.8% Non‐patellar denervation 33.7%	PD can decrease the incidence and severity of AKP within 12 months after TKA, but not after 12‐month follow‐up, no differences in complication and reoperation, better pain relief early postoperative may improve ROM in the PD group after 12‐month follow‐up
Zhou et al. [35]	12 RCTs, 2011–2023	Circumferential patellar denervation 27.5% Non‐patellar denervation: 37.0%	PD is effective in reducing AKP after TKA without PR, both within 12 months and beyond, no increase in complications or reoperations, regarding functional outcome (KSS, OKS, and patellar score), the minimal advantage achievable may not be clinically significant

Abbreviations: AKP, anterior knee pain; AKSKS, American Knee Society Knee Score; AKSFS, American Knee Society Function Score; CR, cruciate retaining; FU, follow‐up; KOOS, Knee Injury and Osteoarthritis Outcome Score; KSS, Knee Society Score; OKS, Oxford Knee Score; PD, patellar denervation; PFS, Patellar Feller Score; PND, patellar non‐denervation; PNR, patella non‐resurfacing; PR, patella resurfacing; PS, posterior stabilised; RCT, randomised controlled trial; ROM, range of motion; TKA, total knee arthroplasty; VAS, Visual Analogue Scale.

There are a variety of factors that contribute to AKP, among them surgical, implant‐ and patient‐related factors. Surgical factors include component malposition, joint line alteration, flexion instability and patellar maltracking, which should be avoided. Patellar denervation and/or lateral facetectomy can reduce AKP, as well as patellar resurfacing [7, 14, 29]. Whether the fad‐pat should be resected or not has not been clearly clarified [7, 23, 29, 34]. Implant factors include various design features. Implants have been considered ‘patella‐friendly’ based on the following design features: [18] relatively posterior flexion‐extension axis which improves extensor mechanism function, lateralized proximal trochlear design, deepened and distally extended trochlear groove and conformity of the anterior flange groove. Despite these design improvements, no relevant reduction in AKP was observed with this ‘patella‐friendly’ TKAs [4]. Currently, there are no conclusive data on whether the bearing type affects AKP [15, 23]. In our cohort, there was less AKP when UC inserts were used instead of PS TKA, but these results were not statistically significant. Patient factors include sociodemographic and psychological factors, which are difficult to modify. Obesity and lower knee function have been associated with AKP [11, 22]. In our patients, higher BMI and younger age was associated with higher risk for AKP. These are well‐known risk factors for AKP and affected patients should be counselled accordingly to avoid unrealistic expectations. Additionally, the severity of functional impairment had an impact on the results after TKA. Poorer preoperative OKS was significantly associated with a higher risk for AKP after one year [6], which is consistent with our cohort. Lower OKS corresponded with more pain and functional limitations which might be caused by more advanced osteoarthritis, central sensitisation or a lower pain threshold.

Considering the high number of patients with some degree of AKP, only a small proportion finally receive secondary patellar resurfacing. A large National Joint Registry (NJR) study, reported an incidence of 0.4% of secondary patellar resurfacing in 536,228 TKAs without patellar resurfacing at first surgery [15]. The cumulative risk of all‐cause revision was 3.5% without and 3.0% with patellar resurfacing. The authors concluded that this represents a substantial healthcare burden and that patellar resurfacing should be considered in primary TKA. Recently, Gunderson et al. [14] reported a rate of 0.4% secondary patellar resurfacing in 1053 TKAs. Despite the incidence for secondary patellar resurfacing was the same as reported in the NJR, the authors conclude, contradictorily, that this excellent result is very promising for the use of modern ‘patella‐friendly’ implants. Since the authors used a protocol for selective patellar resurfacing, patients with risk factors for AKP underwent patellar resurfacing during the initial TKA procedure. These rates are fairly similar to the 0.6% in our patients over a slightly longer observation period. In a meta‐analysis, a rate of 3.9% secondary patellar resurfacing (56 from 1456) was reported from 26 RCTs [24], which is considerably higher, as already mentioned. This raises the question of whether secondary patellar resurfacing is generally performed for clear medical reason or sometimes just because patients continue to have complaints and the patella was not initially resurfaced. The fact that by far not all patients improve after secondary patellar resurfacing suggests that the patella—resurfaced or not—is not always the cause of persistent pain after TKA. A recent meta‐analysis of the outcomes of secondary patellar resurfacing found that PROMs improved in only 53% of patients after this intervention [2]. The authors stated a lack of standardised objective selection criteria for the procedure in the included studies.

Despite lower risk of AKP after patellar resurfacing, there were no differences in PROMs in a recent meta‐analysis of RCTs on primary TKA with or without patellar resurfacing [4, 13, 24, 28]. A systematic review found six RCTs with simultaneous bilateral TKAs in which the patella was resurfaced in only one knee of each patient [5]. The authors included three outdated studies with small numbers of patients and three studies with contemporary implants. Interestingly, the majority of patients were unaware of differences between both knees, nor were there differences in AKP, PROMs or reoperation rates. Although the number of patients in these studies was limited, this is valuable data as the comparison within the same patient and the use of the same implant controls for patient‐ and implant‐related factors that may contribute to AKP.

Despite numerous studies, the literature remains conflicting as to whether the patella should be resurfaced during TKA or not. Resurfacing during primary TKA obviously prevents secondary resurfacing, but adds cost and time to the surgery, which may only be beneficial for a few patients [16]. In particular, it has been reported that the need for secondary patellar resurfacing depends on the specific TKA implants [12, 15]. It seems that lateral facetectomy and denervation in conjunction with ‘patella‐friendly’ implants may be similarly effective [7, 24], but further studies are needed to prove this.

The strengths of the present study include the large sample size, the high follow‐up rate and the detailed assessment of AKP. Limitations include the definition of AKP, which is heterogeneously assessed in the literature. We used a combination of pain questions from validated PROMs and an additional single‐item question on the presence of AKP. This definition still implies a partially subjective assessment, but is as accurate as possible. Radiological outcomes on patella tracking and wear were not investigated. Not all eligible patients could be included as some were lost to follow‐up. However, the follow‐up rate compares favourably with other published studies. In spite of being one of the largest single‐centre series to date, the number of patients was relatively samll to identify factors influencing AKP. Although the surgical procedure of TKA was well standardised, different surgeons used different implants during the study period. No data are available on the progression or regression of AKP over time.

## CONCLUSION

AKP was reported by a relevant proportion of patients after TKA without patellar resurfacing. Affected patients showed worse PROMs and satisfaction. Patients should be counselled accordingly in shared decision making.

## AUTHOR CONTRIBUTIONS

All authors contributed to the study conception and design. Material preparation was performed by Jörg Lützner and Cornelia Lützner, data collection and analysis were performed by Patrick Schubert and Franziska Beyer. The first draft of the manuscript was written by Jörg Lützner and all authors commented on previous versions of the manuscript. All authors read and approved the final manuscript.

## CONFLICT OF INTEREST STATEMENT

The authors declare no conflicts of interest. Outside this work, Jörg Lützner has received institutional research grants from B.Braun Aesculap, Enovis Mathys and ZimmerBiomet as well as honoraria for lectures from B.Braun Aesculap and Enovis Mathys. Jörg Lützner is a member of the Steering Committee of ISAR.

## ETHICS STATEMENT

Ethical approval was granted from the institutional review board (EK 135042014). The study was performed at the University Hospital Carl Gustav Carus, Technische Universität Dresden, Germany. All patients signed informed consent including consent to participate and to publish. All patients signed informed consent.

## Data Availability

The data that support the findings of this study are available from the corresponding author upon reasonable request.

## References

[1] American Joint Replacement Registry (AJRR). Annual Report. 2023. Accessed September 11, 2024. http://www.aaos.org/registries/publications/ajrr-annual-report

[2] Andronic O , Suravaram V , Lu V , Wall SJ , Bucher TA , Prosser GH , et al. What are the outcomes of secondary patella resurfacing for dissatisfaction following primary knee arthroplasty? A systematic review and meta‐analysis of 604 knees. J Arthroplasty. 2024;39:1093–1107.e1. 37871862 10.1016/j.arth.2023.10.027

[3] Beswick AD , Wylde V , Gooberman‐Hill R , Blom A , Dieppe P . What proportion of patients report long‐term pain after total hip or knee replacement for osteoarthritis? A systematic review of prospective studies in unselected patients. BMJ Open. 2012;2:e000435. 10.1136/bmjopen-2011-000435 PMC3289991 22357571

[4] Chen K , Dai X , Li L , Chen Z , Cui H , Lv S . Patellar resurfacing versus nonresurfacing in total knee arthroplasty: an updated meta‐analysis of randomized controlled trials. J Orthop Surg. 2021;16:83. 10.1186/s13018-020-02185-5 PMC7830853 33494774

[5] Choi KY , In Y , Kim MS , Sohn S , Koh IJ . Is the patient aware of the difference between resurfaced and nonresurfaced patella after bilateral total knee arthroplasty? A systematic review of simultaneous bilateral randomized trials. Knee Surg Relat Res. 2022;34:4. 35164884 10.1186/s43019-022-00133-7 PMC8842956

[6] De Oliveira Silva D , Webster KE , Feller JA , McClelland JA . Anterior knee pain following primary unilateral total knee arthroplasty with posterior‐stabilized prosthesis and patellar resurfacing: prevalence and clinical implications. J Arthroplasty. 2023;38:281–285. 36067885 10.1016/j.arth.2022.08.042

[7] Duan G , Liu C , Lin W , Shao J , Fu K , Niu Y , et al. Different factors conduct anterior knee pain following primary total knee arthroplasty: a systematic review and meta‐analysis. J Arthroplasty. 2018;33:1962–1971.e3. 29398258 10.1016/j.arth.2017.12.024

[8] Dutch Arthroplasty Register (LROI). Annual report. 2023. Accessed 11.09.2024. https://www.lroi-report.nl/app/uploads/2023/10/PDF-LROI-annual-report-2023-1.pdf

[9] El‐Othmani MM , Zalikha AK , Shah RP . Anterior knee pain after total knee arthroplasty: a critical review of peripatellar variables. JBJS Rev. 2023;11:e23. 10.2106/JBJS.RVW.23.00092 37478304

[10] Feng H , Feng ML , Cheng JB , Zhang X , Tao HC . Meta‐analysis of factors influencing anterior knee pain after total knee arthroplasty. World J Orthop. 2024;15:180–191. 38464355 10.5312/wjo.v15.i2.180 PMC10921178

[11] Ferreira AS , Mentiplay BF , Taborda B , Pazzinatto MF , de Azevedo FM , de Oliveira Silva D . Overweight and obesity in young adults with patellofemoral pain: Impact on functional capacity and strength. J Sport Health Sci. 2023;12:202–211. 33296724 10.1016/j.jshs.2020.12.002 PMC10105019

[12] German Arthroplasty Registry (EPRD). Annual Report. 2023. Accessed 11.09.2024. https://www.eprd.de/fileadmin/user_upload/Dateien/Publikationen/Berichte/AnnualReport2023-Web_2024-03-26_F.pdf

[13] Grela M , Barrett M , Kunutsor SK , Blom AW , Whitehouse MR , Matharu GS . Clinical effectiveness of patellar resurfacing, no resurfacing and selective resurfacing in primary total knee replacement: systematic review and meta‐analysis of interventional and observational evidence. BMC Musculoskelet Disord. 2022;23:932. 36273138 10.1186/s12891-022-05877-7 PMC9587662

[14] Gunderson ZJ , Luster TG , Deckard ER , Meneghini RM . The fate of unresurfaced patellae in contemporary total knee arthroplasty: early to midterm results. J Arthroplasty. 2024;39:S65–S69. 10.1016/j.arth.2024.01.055 38336307

[15] Hunt LP , Matharu GS , Blom AW , Howard PW , Wilkinson JM , Whitehouse MR . Patellar resurfacing during primary total knee replacement is associated with a lower risk of revision surgery. Bone Joint J. 2021;103–B:864–871. 10.1302/0301-620X.103B5.BJJ-2020-0598.R2 33934661

[16] Kermanshahi N , Budhiparama NC , Wahhab MS , Arias C , Xu W , Schutte D , et al. Should the patella be resurfaced during primary total knee arthroplasty? An updated meta‐analysis and systematic review. J Arthroplasty. 2025;40:102. 39428017 10.1016/j.arth.2024.10.048

[17] National Institute for Health and Care Excellence (NICE). Joint replacement (primary): hip, knee and shoulder (NG 157). 2020. Accessed 15.08.2024. https://www.nice.org.uk/guidance/ng157/chapter/Recommendations#procedures-for-primary-elective-knee-replacement 32881469

[18] Pavlou G , Meyer C , Leonidou A , As‐Sultany M , West R , Tsiridis E . Patellar resurfacing in total knee arthroplasty: does design matter? A meta‐analysis of 7075 cases. J Bone Jt Surg. 2011;93:1301–1309. 10.2106/JBJS.J.00594 21792496

[19] Payne CS , Deckey DG , Verhey JT , Van Schuyver PR , Bingham JS , Spangehl MJ . Global trends in patellar resurfacing from 2004 to 2022. J Arthroplasty. 2025;40:367–372. 39182530 10.1016/j.arth.2024.08.033

[20] Petersen W , Rembitzki IV , Brüggemann GP , Ellermann A , Best R , Koppenburg AG , et al. Anterior knee pain after total knee arthroplasty: a narrative review. Int Orthop. 2014;38:319–328. 24057656 10.1007/s00264-013-2081-4 PMC3923935

[21] Qin Y , Pu C , Zhou Y , Yu J , Tang J . Influence of patellar denervation on anterior knee pain and knee function following total knee replacement: a systematic review and meta‐analysis. ANZ J Surg. 2021;91:E690–E695. 34291537 10.1111/ans.17078

[22] Raud B , Gay C , Guiguet‐Auclair C , Bonnin A , Gerbaud L , Pereira B , et al. Level of obesity is directly associated with the clinical and functional consequences of knee osteoarthritis. Sci Rep. 2020;10:3601. 32107449 10.1038/s41598-020-60587-1 PMC7046749

[23] Simpson CJRW , Wright E , Ng N , Yap NJ , Ndou S , Scott CEH , et al. Patellar resurfacing versus retention in cruciate‐retaining and posterior‐stabilized total knee arthroplasty. Bone Joint J. 2023;105–B:622–634. 10.1302/0301-620X.105B6.BJJ-2022-0970.R2 37257851

[24] Simpson CJ , Ng N , Ndou S , Wright E , Yap NJ , Scott CEH , et al. Patellar resurfacing was not associated with a clinically significant advantage when a modern patellar friendly total knee arthroplasty is employed: a systematic review and meta‐analysis. Knee. 2023;41:329–341. 36827957 10.1016/j.knee.2023.01.021

[25] Smith PNGD , McAuliffe MJ , McDougall C , Stoney JD , Vertullo CJ , Wall CJ , et al. Hip, Knee and Shoulder Arthroplasty: 2023 Annual Report, Australian Orthopaedic Association National Joint Replacement Registry. Adelaide, South Australia: AOA; 2023.

[26] Sun C , Zhang X , Lee WG , Tu Y , Li H , Cai X , et al. Infrapatellar fat pad resection or preservation during total knee arthroplasty: a meta‐analysis of randomized controlled trials. J Orthop Surg. 2020;15:297. 10.1186/s13018-020-01823-2 PMC7409474 32758250

[27] Swedish Arthroplasty Register (SAR). Annual Report. 2023. Accessed 11.09.2024. https://registercentrum.blob.core.windows.net/sar/r/SAR_Annual-report-2023_EN-DS5gryeOB.pdf

[28] Tang X , He Y , Pu S , Lei L , Ning N , Shi Y , et al. Patellar resurfacing in primary total knee arthroplasty: a meta‐analysis and trial sequential analysis of 50 randomized controlled trials. Orthop Surg. 2023;15:379–399. 36479594 10.1111/os.13392 PMC9891932

[29] van Jonbergen HPW , Reuver JM , Mutsaerts EL , Poolman RW . Determinants of anterior knee pain following total knee replacement: a systematic review. Knee Surg Sports Traumatol Arthrosc. 2014;22:478–499. 23160846 10.1007/s00167-012-2294-x

[30] Walker H , Rao A , Tsimiklis J , Smitham P . Are short term outcomes superior following total knee arthroplasty when infra‐patellar fat pad is resected? A systematic review and meta‐analysis. ANZ J Surg. 2024;94:1234–1239. 38982806 10.1111/ans.19148

[31] Wang Y , Feng W , Zang J , Gao H . Effect of patellar denervation on anterior knee pain and knee function in total knee arthroplasty without patellar resurfacing: a meta‐analysis of randomized controlled trials. Orthop Surg. 2020;12:1859–1869. 33112040 10.1111/os.12815 PMC7767783

[32] Watson CJ , Propps M , Ratner J , Zeigler DL , Horton P , Smith SS . Reliability and responsiveness of the lower extremity functional scale and the anterior knee pain scale in patients with anterior knee pain. J Orthop Sports Phys Ther. 2005;35:136–146. 15839307 10.2519/jospt.2005.35.3.136

[33] Wylde V , Beswick A , Bruce J , Blom A , Howells N , Gooberman‐Hill R . Chronic pain after total knee arthroplasty. EFORT Open Rev. 2018;3:461–470. 30237904 10.1302/2058-5241.3.180004 PMC6134884

[34] Yuan M , Ding Z , Ling T , Zhou Z . Patellar denervation with electrocautery reduces anterior knee pain within 1 year after total knee arthroplasty: a meta‐analysis of randomized controlled trials. Orthop Surg. 2021;13:14–27. 33354916 10.1111/os.12735 PMC7862158

[35] Zhou X , Jiang Y , Chen D , Chen T , Tian Z . Does patellar denervation with electrocautery benefits for total knee arthroplasty without patellar resurfacing: a meta‐analysis of randomized controlled trails. Orthop Surg. 2024;16:1832–1848. 38951735 10.1111/os.14161 PMC11293931

